# New platform for simple and rapid protein-based affinity reactions

**DOI:** 10.1038/s41598-017-00264-y

**Published:** 2017-03-14

**Authors:** Kei Kubota, Takuya Kubo, Tetsuya Tanigawa, Toyohiro Naito, Koji Otsuka

**Affiliations:** 1 Graduate School of Engineering, Kyoto University, Kyoto, Japan; 2 Analytical and Quality Evaluation Research Laboratories, Daiichi Sankyo Co., Ltd., Hiratsuka, Japan; 3 Chemco Scientific Co., Ltd., Osaka, Japan

## Abstract

We developed a spongy-like porous polymer (spongy monolith) consisting of poly(ethylene-co-glycidyl methacrylate) with continuous macropores that allowed efficient *in situ* reaction between the epoxy groups and proteins of interest. Immobilization of protein A on the spongy monolith enabled high-yield collection of immunoglobulin G (IgG) from cell culture supernatant even at a high flow rate. In addition, immobilization of pepsin on the spongy monolith enabled efficient online digestion at a high flow rate.

## Introduction

A variety of antibody-based medicines have been approved in recent years^1, 2^. These products have high annual returns due to their high selectivity toward their target antigens, relatively low levels of side effects, and stability *in vivo*; in addition, these medicines can be produced using standard cell culture procedures^3–7^. To obtain a high-quality antibody medicine at low cost, it is necessary to select highly productive cells, optimize the culture conditions, and develop an efficient purification method. To evaluate the productivity of a system for biosynthesis of an antibody, especially of the immunoglobulin G (IgG) subtype, a chromatographic system using a protein A immobilized column is often employed for selection and optimization of the cell culture. In order to process a large number of samples, it is necessary to perform rapid optimization using high-throughput chromatography^8, 9^. Indeed, for certain antibodies, more than 100 kg is required at the clinical investigation stage^10–12^. Usually, separation media in which protein A is immobilized onto a crosslinked-agarose adsorbent are used for the analysis and purification of an antibody^13–15^ as a suitable separation antibodies^16–19^. However, as for currently available separation media, elution throughput is often limited, resulting in an inefficient optimization of purification and productivity. Furthermore, the expense of such adsorbents (30-fold higher than other typical adsorbents) and the difficulty of column packing contribute to the high final price of antibody-based medicines^20–22^. Therefore, there is an urgent demand for new separation media that can facilitate higher throughput and lower cost.

To achieve high throughput and low cost, monolithic materials with continuous three-dimensional (3D) structures are advantageous^23–25^. For purification of biomolecules, the monolithic structure is suitable for rapid reactions because the flow-through pores are integrated with the skeleton^26–31^. In a typical monolith, silica- or polymer-based materials are prepared by sol–gel reaction and/or phase separation. Accordingly, the control of pore size, especially for larger pore (>10 μm), scale up in column size, and packing to columnar tubes are not easy. Instead of these typical monoliths, we proposed using a sponge-like material or spongy monolith as a novel separation medium^32, 33^. The spongy monolith is prepared simply by blending a thermoplastic resin above its melting point with water-soluble pore templates. After removal of the pore template by washing with water, the resultant spongy monolith contains large flow-through pores of >10 μm in diameter, and a column made of the spongy monolith facilitates separation mediated by hydrophobic interactions and/or ion exchange at a high flow rate. Additionally, the spongy monolith can be prepared in any shapes and easily packed into a column. Therefore, we expected that the spongy monolith containing specific functional groups, such as epoxy groups, would be useful for affinity chromatography and overcome the limitations of current media.

## Results and Discussion

In this study, we prepared a novel spongy monolith consisting of poly(ethylene-co-glycidyl methacrylate) (PEGM). After the monolith was packed into a column, protein A was immobilized onto the media *in situ*, and the affinity reaction was quantitatively examined and validated under high-throughput conditions. In an additional application of the new platforms, we immobilized the digestive enzyme pepsin onto the spongy monolith and performed online flow digestion of an antibody, and then determined the primary structure of the antibody from the peptide fragments.

**Figure 1:**
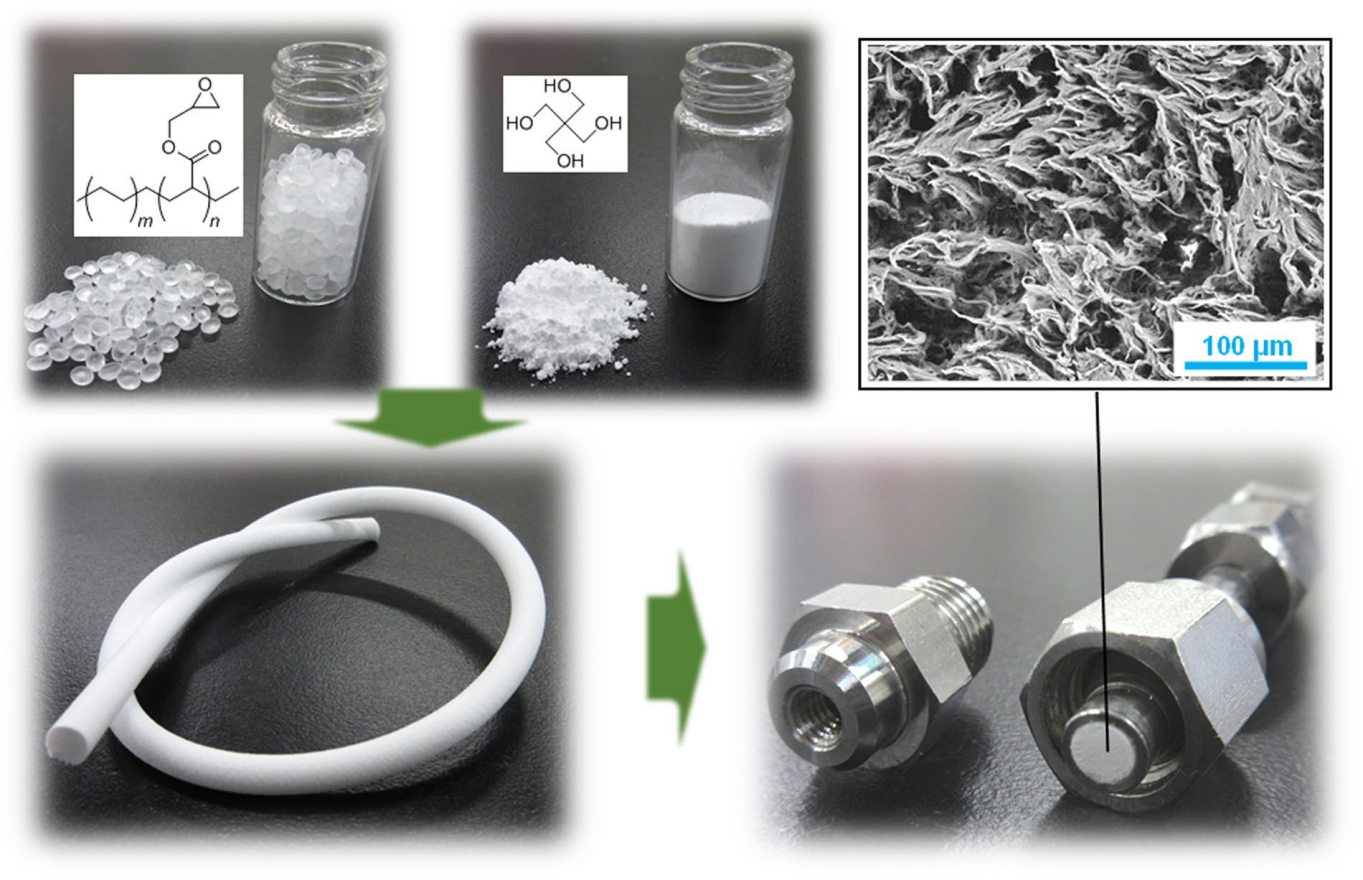
Protein A immobilized spongy monolith (ProA-SpM). Physical appearances of Pro-SpM including primary materials, a molded item, and a packed column.

**Figure 2:**
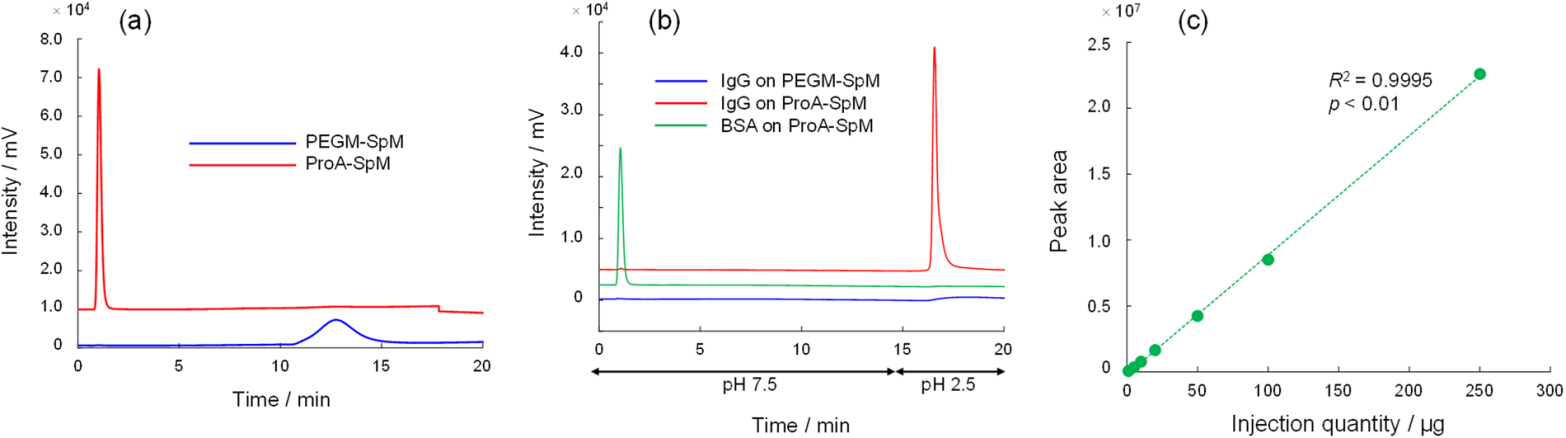
UV Chromatograms of IgG and BSA on the PEGM-SpM and ProA-SpM. (**a**) Reversed-phase chromatograms of IgG-1 with the PEGM-SpM and ProA-SpM. (**b**) Affinity separation of IgG-1 by a stepwise pH gradient. (**c**) A relation between the injected amount of IgG-1 and the peak area with the ProA-SpM.

As shown in Fig. 1, a PEGM spongy monolith (PEGM-SpM) was successfully prepared with the expected morphology. In brief, the average pore size of the prepared PEGM-SpM was ~10 μm, as determined by mercury porosimeter (Supplementary Fig. 1), whereas no meso-pores were detected by nitrogen-gas adsorption analysis. The PEGM-SpM was packed into a stainless-steel column by a simple method (Supplementary Fig. 2) similar to that used in our previous study. After the column was conditioned with methanol and water, a protein A solution (1.0 mg mL^−1^ in PBS) was passed through the column, and then incubated at 37 °C for 16 h after both ends of the column were sealed. No morphological alteration was observed following the protein A modification (Supplementary Fig. 3). To confirm the effect of the modification, we then analyzed the column (ProA-SpM) by liquid chromatography (LC). As shown in Fig. 2.(a), IgG-1 was strongly retained on the original PEGM-SpM via hydrophobic interaction in a typical reversed-phase LC (RPLC) mode. On the other hand, the ProA-SpM exhibited significantly less hydrophobic interaction, resulting in faster elution of IgG-1. A similar phenomenon was observed when BSA was used as a solute (Supplementary Fig. 4). These results indicated that protein A effectively covered the skeleton surface of the monolith, dramatically suppressing non-selective hydrophobicity. Next, we confirmed the affinity of the ProA-SpM via simple pH-gradient LC, which is commonly employed to evaluate protein A-immobilized columns because the interaction between protein A and IgG occurs only at pH >7. The resultant chromatograms are summarized in Fig. 2.(b). As expected, IgG-1 was selectively retained on the ProA-SpM and released by a one-step pH gradient. By contrast, BSA was quickly eluted without any retention, and PEGM-SpM adsorbed IgG-1 due to its high hydrophobicity. Additionally, another IgG family member, IgG-2, was also effectively separated on the ProA-SpM (Supplementary Fig. 5). In a simplified validation, we injected various amounts of IgG-1 into the ProA-SpM, and found that the linear range of peak area was at least 1.0–250 µg (Fig. 2.(c)). In addition, we evaluated the accuracy by continuous analyses (*n* = 6), and estimated the relative standard deviation (RSD) as 0.7%. According to these results, this simply prepared ProA-SpM column had affinity similar to that of commercially available protein A-immobilized columns.

To be most useful, an affinity column must have abundant adsorption capacity for the ligand(s) of interest. To evaluate the maximum adsorption capacity due to immobilized protein A, we performed a frontal analysis, a method commonly utilized to evaluate capacity by LC^34, 35^, of both our column and a commercially available protein A-immobilized column (ProA-Column). This analysis revealed that the densities of immobilized protein A in the ProA-SpM and ProA-Column were 1.0–4.2 nmol g^−1^ and 5–21 nmol g^−1^, respectively (Supplementary Fig. 6). Although the density of immobilized protein A was slightly lower in the ProA-SpM, the adsorption capacity for IgG-1 was comparable between the two columns (0.31 mg for the ProA-SpM and 0.32 mg for the ProA-Column). Therefore, our ProA-SpM had sufficient capacity to serve as an affinity column for the effective separation of IgG.

**Figure 3:**
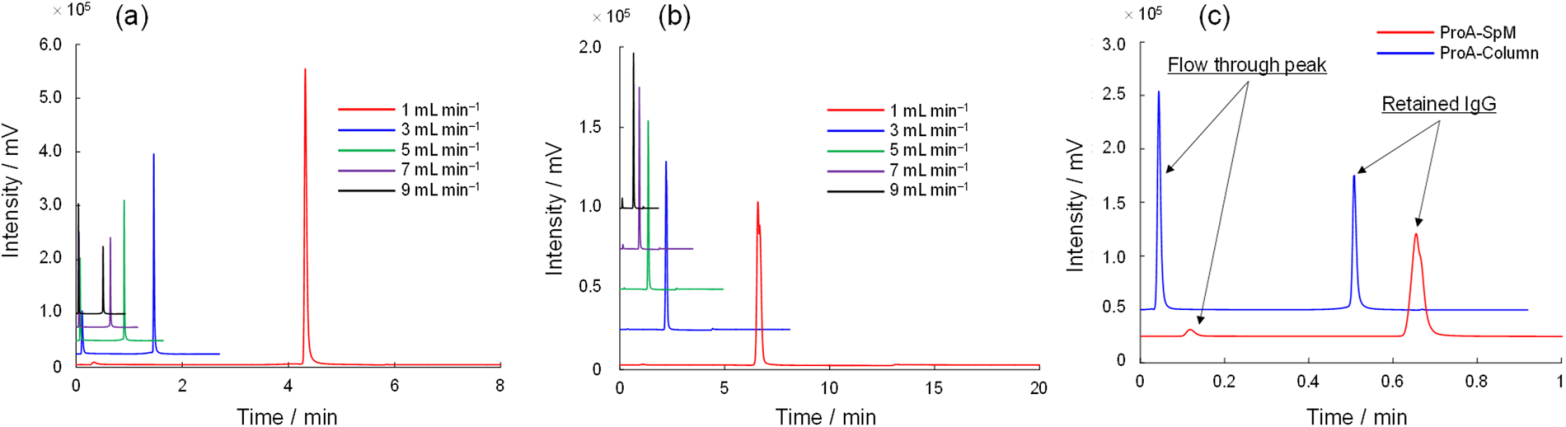
The UV chromatograms for IgG on protein A immobilized column under various flow rates. Affinity separations with a various flow rates with the ProA-Column (**a**) and ProA-SpM (**b**). (**c**) Rapid separation of IgG from a protein A load sample with the ProA-Column or ProA-SpM at 9.0 mL min^−1^.

**Figure 4:**
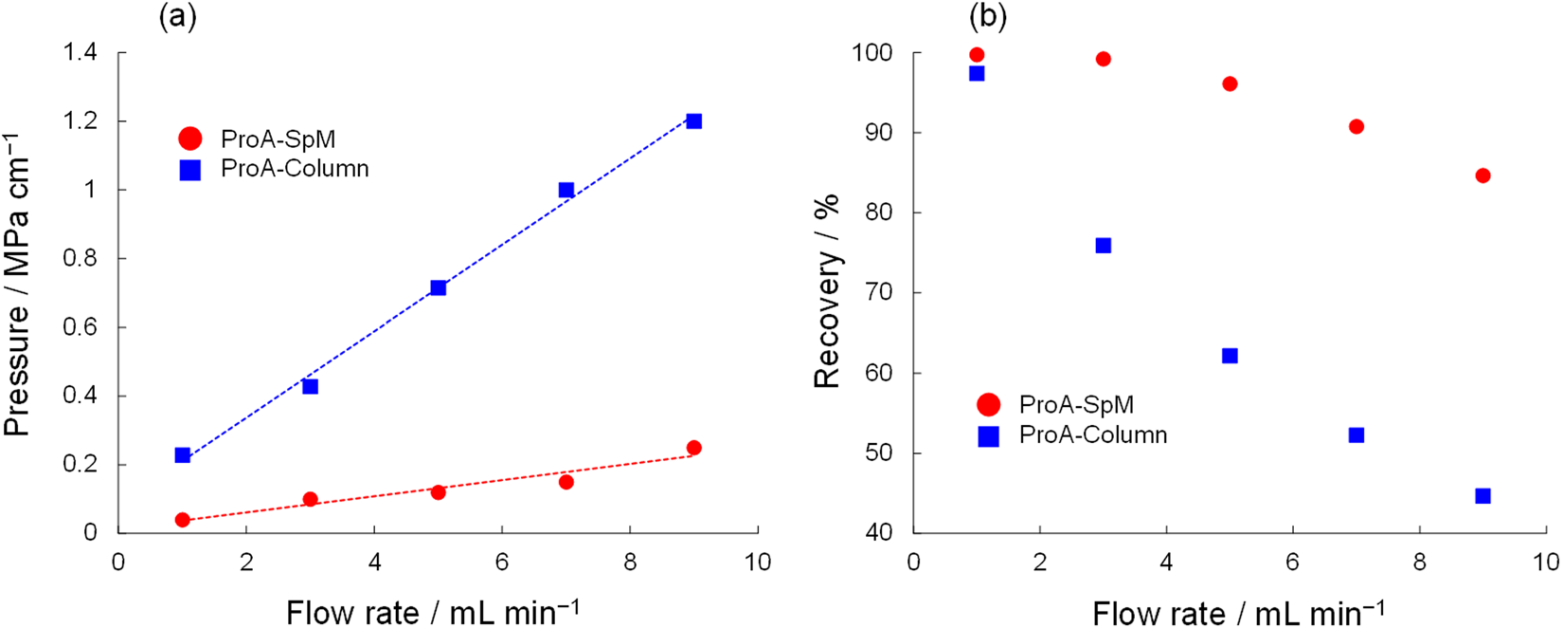
Back pressure and recovery of IgG on the ProA-Column and ProA-SpM. (**a**) Comparison of back pressure on LC eluted by 50 mM phosphate buffer with 150 mM NaCl as a mobile phase using the ProA-Column or ProA-SpM. (**b**) Comparison of the recovery for IgG with the ProA-Column or ProA-SpM, total amount was estimated by sum of the peak area among the flow though peak and IgG.

The most important advantage of the spongy monolith is its potential for high-throughput elution. To evaluate this feature, we carried out a similar affinity separation using IgG-1 as the solute under various flow rate conditions; the results are summarized in Fig. 3. When the ProA-Column was utilized at a higher flow rate, the flow-through fraction was presented in front of the solvent peak, as shown in Fig. 3(a). By contrast, the ProA-SpM allowed higher recovery, even at a high flow rate. Figure 3(c) shows the chromatograms for both the ProA-Column and ProA-SpM at a flow rate of 9.0 mL min^−1^. Obviously, the collected and flow-through peaks were completely different from each other. The backpressure and recovery of IgG on the columns at each flow rate are summarized in Fig. 4(a) and (b), respectively. For the ProA-SpM, both backpressure and recovery were superior to those of the ProA-Column. A potential reason for these significant differences, especially in recovery at a high flow rate, is that the affinity interaction between a protein-based ligand and an antibody under a higher flow rate is generally not effective in a column in which spherical and porous beads are packed, due to the lower accessibility caused by slower mass transfer. Usually, in an LC analysis, the van Deemter equation (1)^36^ is employed to determine the plate height, *H*, which is defined as diffusion per column length and directly contributes to the separation efficiency:

1 $$H=A\,{d}_{P}+(B\,{D}_{m})/u+(C\,{d}_{P}^{2}u)/{D}_{m}$$

Here, *d*_P_, *D*_m_, and *u* are the diameter of the packed particle, diffusion coefficient of the solute, and linear velocity, respectively, and *A*, *B*, and *C* are constants corresponding to eddy diffusion, longitudinal diffusion, and mass transfer, respectively. Under higher linear velocity, mass transfer is usually predominant, resulting in low separation efficiency. For interactions among macromolecules (*e.g.*, protein–protein interactions), slow mass transfer may provide fewer chances for encounter; thus, most of the IgG was eluted without interaction at high flow rates on the ProA-Column. On the other hand, the spongy monolith contains only macro-size flow-through pores, and protein A should be immobilized only on the surface of the monolithic skeleton. Therefore, we anticipated that the ProA-SpM would allow effective interaction between protein A and IgG-1. Additionally, the linearity of recovery at a higher flow rate (9.0 mL min^−1^) is similar to that at a lower flow rate (Supplementary Fig. 7). Furthermore, we investigated the ruggedness of the ProA-SpM. As a result of 100 times repeated analyses with IgG under 9.0 mL min^−1^, the RSDs of the retention time and recovery of IgG were estimated as 0.45% and 0.41%, respectively. Also, the recovery of IgG was kept over 99% even after washing with 0.1 M aqueous NaOH (5 times), which is commonly used as an evaluation for the ruggedness of affinity columns. These results suggested that the ProA-SpM had enough ruggedness as an affinity column. According to these results, we anticipate that the ProA-SpM could be used as a novel affinity separation medium for high-throughput purifications.

**Figure 5:**
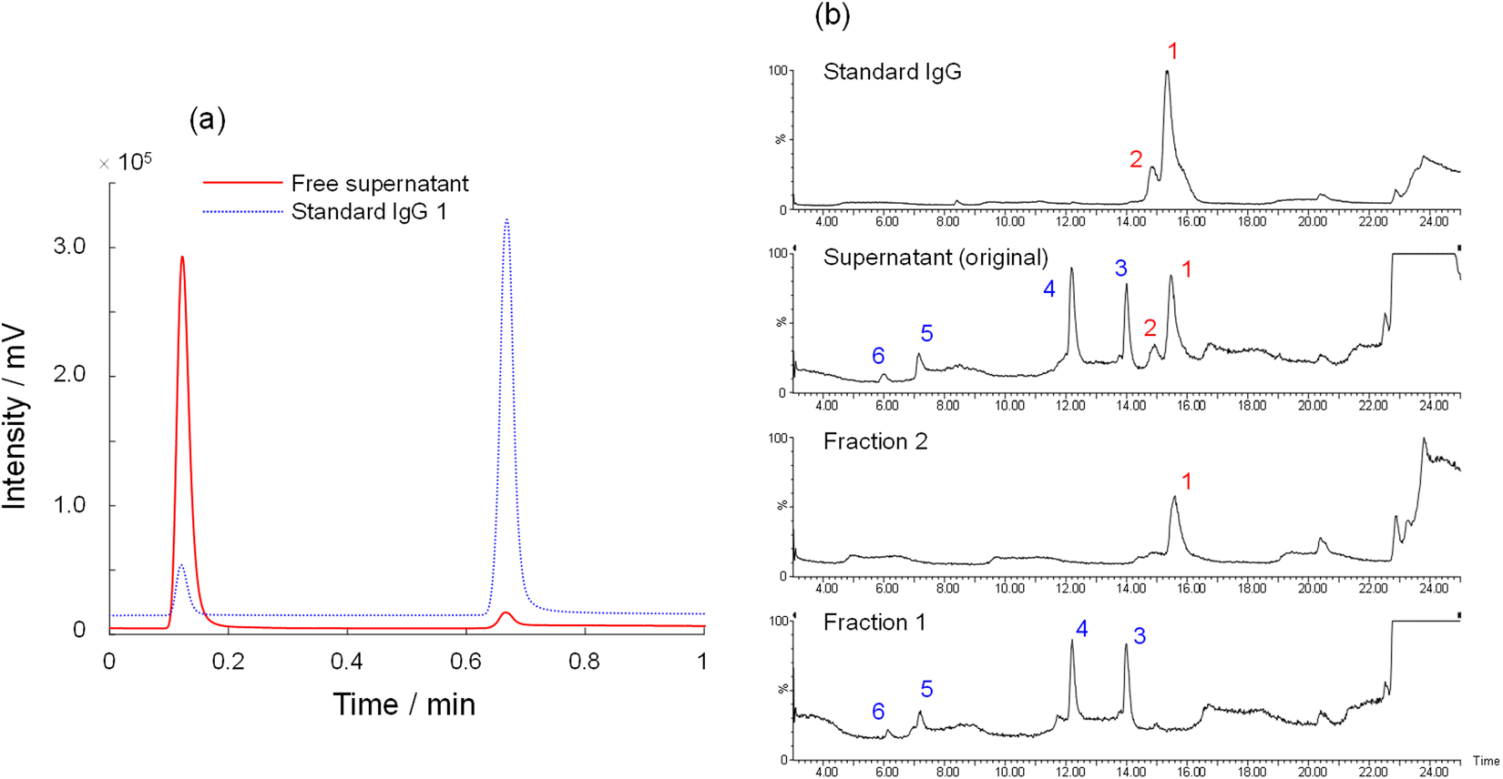
Affinity separation of cell supernatant for isolation of IgG. (**a**) UV chromatograms of a supernatant in a protein A load sample and a standard IgG 1 with the ProA-SpM. (**b**) Total ion chromatograms in RPLC.

To demonstrate purification of IgG from cell culture samples, we used the ProA-SpM. Cell culture supernatant treated using typical procedures was separated with a simple pH gradient using a variety of flow rates. Then, the peak likely to contain IgG was manually fractionated, and the chromatograms of free supernatant and a standard IgG are shown in Fig. 5(a). To confirm the presence of IgG in the fraction, the collected sample was analyzed by authentic RPLC with time-of-flight (TOF) mass spectrometry (MS) (TOF-MS), which is usually employed for the proteins separation using a reversed-phase column with MS detection. As shown in the obtained chromatograms (Supplementary Fig. 8) generated by UV detection, both the supernatant and the first fraction contained a major peak and a few minor peaks, whereas the collected fraction clearly contained only one peak, which corresponded to the standard IgG peak. A comparison of total ion chromatograms is provided in Fig. 5(b). Similar to the UV chromatograms, the collected sample exhibited a clear peak without any other minor peaks. After deconvolution of the original MS results, all the peaks from the original supernatant could be assigned, and the observed MS numbers are summarized in Table 1. In addition, the MS spectra of each peak are described in Supplementary Fig. 9. These results indicate that the collected fraction contained the selectively separated IgG moiety from the natural cell culture sample. Finally, the concentration of IgG in the supernatant of the cell culture was estimated as 0.42 mg mL^−1^. Thus, we successfully demonstrated affinity separation based on the protein A–IgG interaction at high throughput using the newly developed spongy monolith. The method exhibited a good reproducibility and efficient recovery over a wide range of concentrations.

**Table 1:** Peak identifications from a protein A load sample.

Sample	Peak No.	Retention Time (min)	Observed mass (Da)
Standard IgG	1	15.35	149207
	2	14.81	23382
			101380
			125840
Supernatant (original)	1	15.46	149204
	2	14.93	23384
	3	14.00	46894
	4	12.18	23567
	5	7.15	11666
	6	5.99	—
Fraction 2 (IgG fraction from ProA-SpM)	1	15.61	149208
Fraction 1 (flow through fraction from ProA-SpM)	3	13.99	46893
	4	12.21	23566
	5	7.21	11677
	6	6.14	—

Fractions 1 and 2 were collected from the separation (Fig. 1(j)) from the front and back peaks, respectively. The peaks are corresponding to Fig. 1(k). The observed mass was estimated by a deconvolution of multi ions with MaxEnt1.

**Figure 6:**
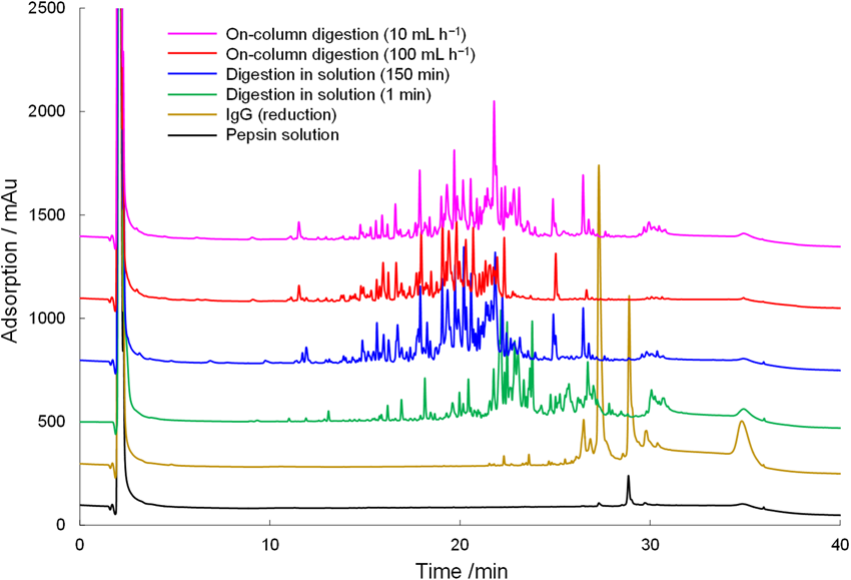
Online digestion of a recombinant human IgG1 antibody with the Pep-SpM. UV chromatograms of the digested antibody in solution or online with the Pep-SpM.

**Figure 7:**
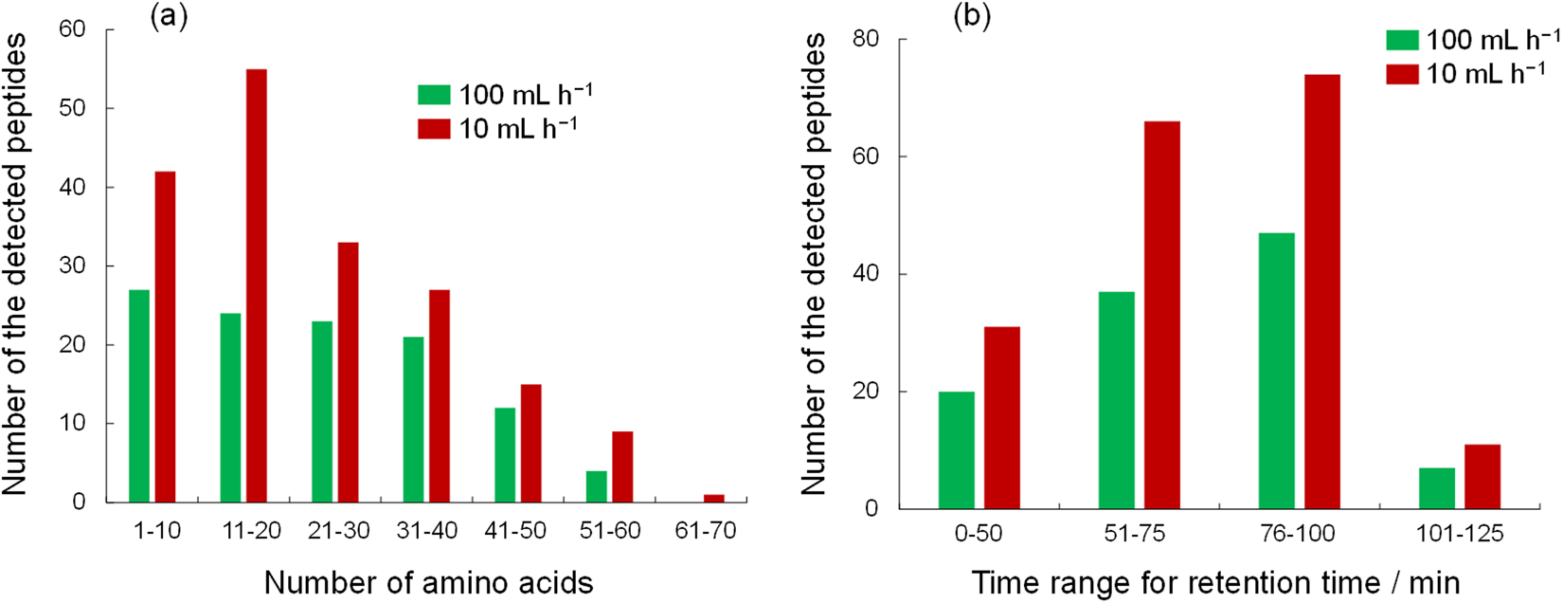
Detected peptides by the online digestion of an antibody by the Pep-SpM. (**a**) The number of the assigned peptides against the number of the composed amino acids. (**b**) The number of the assigned peptides against the elution time in LC separation.

**Figure 8:**
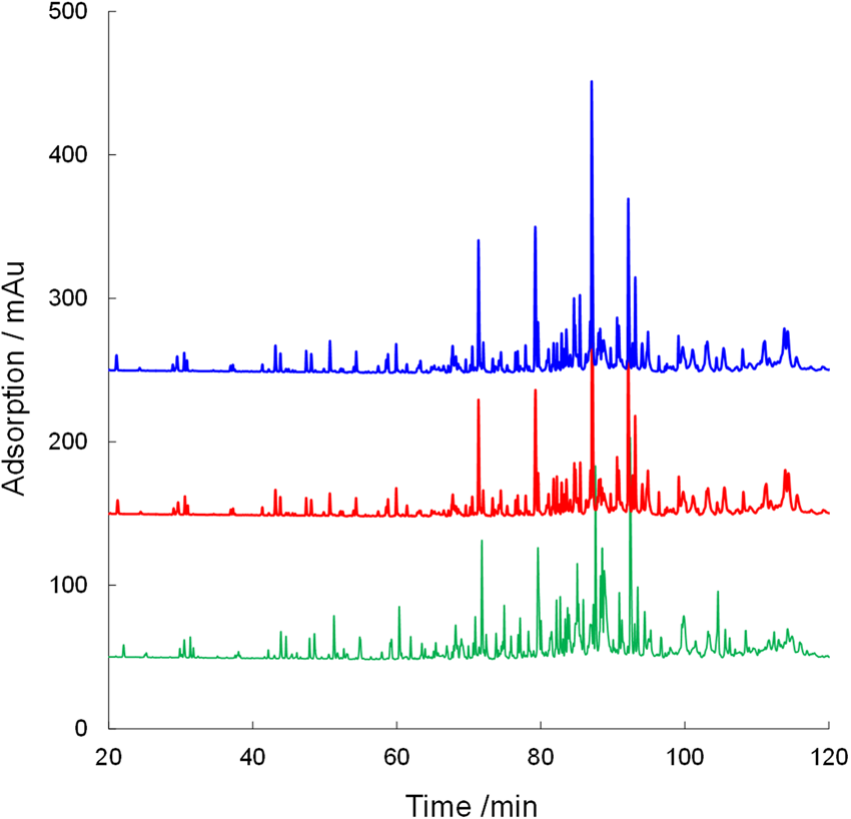
The UV chromatograms for repeatability of online digestion with the Pep-SpM under 10 mL h^−1^ as flow rate.

As mentioned above, the newly developed spongy monolith was suitable for a high-throughput affinity reaction. Because the immobilization of the proteins was based on a simple reaction with epoxy groups in the monolith, we believe that this material could be used for a variety of protein-based reactions. To explore this idea further, we carried out high-throughput online digestion using a digestive enzyme, pepsin. The immobilization of pepsin was performed successfully by a method similar to the one used for protein A. Pepsin is an aspartic protease that cleaves peptide bonds between hydrophobic and preferably aromatic amino acids, such as phenylalanine, tryptophan, and tyrosine. In this evaluation, an antibody solution was introduced into the pepsin-immobilized spongy monolith (Pep-SpM), and the eluted fraction was analyzed by LC–MS. For comparison, samples in a simple solution containing pepsin and the antibody were also analyzed to confirm the cleaved peptides. The UV chromatograms are summarized in Fig. 6. As expected, longer reaction in solution yielded larger peptide fragments. On the contrary, in online digestion with the Pep-SpM, the peptide fragments were much larger, even though a faster flow rate (100 mL h^−1^) was employed. When a slower flow rate (10 mL h^−1^) was used, the detected peaks were almost the same as those in solution samples treated for 150 min. The numbers of peptides detected in LC–MS, as a function of the number of amino acids and elution time, are summarized in Fig. 7. Both figures demonstrate that a slower flow allowed for more extensive digestion. These results clearly showed that effective cleavage occurred in the Pep-SpM. To confirm the sequence of each peptide, a quantitative analysis was also carried out. The theoretical alignment of amino acids in the antibody and the theoretical pepsin digestion fragments (digestion sites: N terminal, F, I, M, Y, W, V; C terminal, C, D, E, F, L, M, T, W, Y) were compared against results generated by the PepFinder 2.0 based on the LC–MS data. All assignment data are summarized in Supplementary Table 1. Coverage for the primary amino acid alignment, as determined from those results, is shown in Table 2. In both elution flows, all 213 residues in the light chain were detected. Additionally, coverage of the heavy chain corresponded to the flow speed; *i.e.*, a slower flow provided higher coverage. These results also supported the idea that efficient online digestion occurred in the Pep-SpM. Finally, we showed that the reproducibility of the online digestion was satisfactory at both flow rates, as shown in Fig. 8 and Supplementary Fig. 10. All these results indicate that the spongy monolith can be used for the effective online digestion.

**Table 2:** Identified amino acids from a recombinant human IgG1 antibody using the Pep-SpM.

	100 mL h^−1^	10 mL h^−1^
Light chain (213 residues)	100% (213/213)	100% (213/213)
Heavy chain (449 residues)	45.7% (205/449)	82.4% (370/449)

The ratio of the detected amino acids was estimated from the all the results of peptides mapping by PepFinder 2.0.

## Conclusion

In summary, we proposed a new platform for protein-based affinity reaction. A spongy monolith containing epoxy groups was effectively used in affinity separation with protein A and digestion with pepsin. Both results demonstrated the utility of the new platform for rapid-flow affinity reactions. We believe that this new platform will be useful for variety of protein-based reactions with rapid flow rates and low costs. Additionally, the platform can be easily scaled up, and we anticipate that future efforts will contribute to purification of antibody-based medicines at the plant level.

## Methods

### Preparation of a spongy monolith

35 w% of poly(ethylene-co-glycidyl methacrylate) (PEGM), in which glycidyl methacrylate of 8% is contained, 52 w% of pore templates (pentaerythritol), whose particle size in diameter was classified around 10 μm, and 7 w% of auxiliary of pore templates (poly(oxyethylene, oxypropylene)) triol were melted at 130 °C and homogeneously kneading. The resulting material was extruded as a columnar shape at 130 °C. The columnar material was immediately cooled in water to obtain the stick like material. After cooling, the material was washed in water under an ultrasonication to remove water-soluble compounds. At this stage, water-soluble compounds functioned as the pore templates. The porosity of the spongy monolith calculated by a void volume on LC was 65% and the diameter of its cross section across its entire length was 4.8 mm. (PEGM-SpM).

### Packing of a spongy monolith

For packing spongy monoliths in a stainless steel column, we utilized an empty column with an internal diameter of 4.6 mm (Supplementary Fig. 2). The diameter of the spongy monolithic column was greater than the internal diameter of the empty column (4.6 mm). Nevertheless, the elasticity of the spongy monolith material facilitated the packing. The procedure for packing was as follows: One end of the spongy monolith was compressed with a thermal shrinkage tube at 120 °C. After cooling, the shrinkage tube was removed; and the diameter of the compressed end of the spongy monolith was reduced less than 4.6 mm. After macerating the spongy monolith into ethylene glycol as a lubricity agent, the shrunk portion of the spongy monolith was inserted into the empty column and pulled from the other end, until the non-shrunk portion completely filled the column. Finally, the excess portion of the spongy monolith was cut and the column end module was connected. At this point, the shrunken end of the spongy monolith was completely cut and only the portion of the material with the initial diameter was packed into the column. Then, the prepared column was connected to a pump of LC for continuous elution. The mixture of methanol/water was eluted to the column for further washing to remove the pore templates and the homogenization of the packing^37, 38^ condition.

### Preparation of a protein A immobilized column

Phosphate buffered salts (PBS) solution was prepared with a PBS tablet into pure water of 100 mL (9.57 mM, pH 7.5). Pierce Recombinant Protein A of 5 mg was dissolved in the PBS solution of 5 mL. For conditioning the column, acetonitrile (MeCN) and pure water were passed through the PEGM-SpM at room temperature for 5 mL in each solvent. The protein A solution (1 mg mL^−1^) was fulfilled into the PEGM-SpM completely, and then the column was incubated at 37 °C for 16 h. The completed column was washed with pure water for 1 h at 1 mL min^−1^. (ProA-SpM).

### Preparation of a pepsin immobilized column

Pepsin of 15 mg was dissolved into 5 vol% formic acid aqueous solution of 5 mL. For conditioning the column, MeCN and pure water were passed through the PEGM-SpM at room temperature for 5 mL in each solvent. The pepsin solution (3 mg mL^−1^) was fulfilled into the PEGM-SpM completely, and then the column was incubated at room temperature for a week. The completed column was washed with 5 vol % formic acid aqueous solution for 1 h at 1 mL min^−1^. (Pep-SpM).

### Conditions for RPLC

For RPLC evaluations, a linear gradient was employed using 0.1 vol% trifluoroacetic acid (TFA) aqueous solution (A) and 0.1 vol% TFA in MeCN at 1.0 mL min^−1^ under 40 °C. The gradient condition was utilized at 100% A to 100%B for 20 min, and 100%B for 20 to 30 min. For an affinity separation of the ProA-SpM, a 50 mM phosphate buffer with 150 mM NaCl pH 7.5 (A) and pH 2.5 (B) was employed at 25 °C. Regarding Fig. 1(b), a linear gradient was utilized at 100% A (0 to 5 min), 100% A to 100% B (5 to 15 min), and 100% B (15 to 25 min). For the other figures, the stepwise gradient was employed. The condition at 1.0 mL min^−1^ was 100% A (0–2.4 min) and 100% B (2.41 to 9.6 min). The gradient conditions were optimized in response to the flow rate.

### Fractionation and determination of IgG from cell culture

To know the possibility for the affinity separation with the ProA-SpM, the real sample was utilized for the separation. Protein A load sample, which was obtained just by simple filtration with membrane filter (0.2 μm) to remove the cells, was directly injected into the ProA-SpM with the same conditions as above. The peak of the seemed to IgG was manually collected (Fraction 2) and the flow through fraction was also collected (Fraction 1). Both fractions, the original supernatant, and a standard IgG were analyzed by LC with a TOF-mass spectrometer. For the intact-MS analysis, the samples were separated with RPLC using an LC-20 Prominence XR (Shimadzu) employing an Aeris Widepore XB-C8 300 Å 2.1 × 100 mm, 3.6 μm column (Phenomenex). The mobile phase A was water/TFA (1000/1) and mobile phase B was water/MeCN/IPA/TFA (100/200/700/1). A linear gradient was set as (Time/B%) = (0/21), (3/21), (21/36), (21.01/100), (25/100), (25.01/21), (35/21) at the column temperature of 85 °C. The flow rate was 0.2 mL min^−1^ and UV detection was carried out at 214 nm. The separated peaks were detected with a Q-Tof premier (Waters), equipped with an electrospray ion source set in the positive ion mode for the m/z of 1000 to 4000. The parent molecular weights were estimated by a deconvolution of multi ions with MaxEnt1^39, 40^ (Waters).

### Online digestion of an antibody by the Pep-SpM and LC–MS analysis for peptide mapping

A reduced antibody sample was prepared with 0.05 M acetic acid and 0.05 M tris(2-carboxyethyl) phosphine hydrochloride in aqueous solution (the concertation of the antibody, 10 mg mL^−1^), and then the solution was stirred at 75 °C for 15 min. The antibody solution was passed through the Pep-SpM at 10 or 100 mL h^−1^, and the eluted solution was collected during every 1 min. On the other hand, the reduction antibody solution was also reacted with a pepsin solution as the comparison for 1 or 150 min. Both the collected fraction by online digestion in the Pep-SpM and by treating in solution were analyzed typical LC. LC conditions are follows; column, AdvanceBio PeptideMap 2.1 × 150 mm; mobile phase, 0.1 vol% TFA in water as mobile phase A and 0.1 vol% TFA in MeCN, 0 to 55% B for 0 to 30 min, 100% B for 30.1 to 40 min; temperature, 50 °C; flow rate, 0.2 mL min^−1^, which is corresponding to Fig. 2(a) using UV detection. For LC–MS analyses, instead mobile phase condition was follows; 0.1 vol% TFA in water as mobile phase A and 0.1 vol% TFA in 90% MeCN aqueous, 0 to 43% B for 5 to 120 min, 100% B for 120.1 to 135 min, which is corresponding to Fig. 2(b,c) and Supplementary Table 1 obtained by PepFinder 2.0. The separated peaks were detected by a mass spectrometer, LTQ/XL Orbitrap (Thermo Fisher Scientific), equipped with an electrospray ion source set in the positive ion mode for the *m/z* of 300 to 2000. MS/MS fragmentation analysis was conducted by using following conditions: the parent ions were fragmented with HCD at the isolation width of 6.0 Da and the collision energy of 35 V. Supplementary Table 1 indicates all the peptides detected by LC–MS containing the origin (light or heavy chain), the number of amino acid of each terminal, and the length. Here, the number of amino acid was assigned that the number 1 is first amino acid from the *N*-terminal, and the total number of amino acids are 213 and 449 in light chain and heavy chain^41, 42^, respectively.

## Electronic supplementary material

Supplementary Information
