# Supplementary material for: New platform for simple and rapid protein-based affinity reactions
Source: Sci Rep. 2017 Mar 14;7:178. doi: 10.1038/s41598-017-00264-y (PMC5428043; doi:10.1038/s41598-017-00264-y)
Supplement: Supplementary file 1 — Supplementary Information [file 41598_2017_264_MOESM1_ESM.pdf]

Supplementary Information

New platform for simple and rapid protein-based affinity reactions

Kei Kubota, Takuya Kubo, Tetsuya Tanigawa, Toyohiro Naito & Koji Otsuka

Materials

Poly(ethylene-co-glycidyl methacrylate) (Sigma Aldrich), pentaerythritol (Toso), recombinant human IgG1 antibody (Daiichi-Sankyo), bovine serum albumin (BSA) (Sigma Aldrich), IgG1, kappa from human myeloma plasma (Sigma Aldrich), IgG2, kappa from human myeloma plasma (Sigma Aldrich), pierce recombinant Protein A (Life Technologies), PBS Tablets (TaKaRa), acetonitrile, HPLC grade (Wako Pure Chemical Industries), acetonitrile, LC-MS grade (Wako Pure Chemical Industries), sodium dihydrogenphosphate dihydrate (Wako Pure Chemical Industries), sodium chloride (Wako Pure Chemical Industries), 2 mol/L sodium hydroxide (Wako Pure Chemical Industries), 2 mol/L hydrochloric acid (Wako Pure Chemical Industries), Pepsin from porcine gastric mucosa (Sigma Aldrich), acetic acid (Wako Pure Chemical Industries), tris(hydroxymethyl)aminomethane (Wako Pure Chemical Industries), formic acid (Wako Pure Chemical Industries), trifluoroacetic acid (TFA) (Wako Pure Chemical Industries). Pure water was obtained from Milli-Q Gradient A10 (Merck Millipore).

Instruments

Scanning electron microscope, TM-1000 (Hitachi High-Technologies). LC analyses for IgG with PDA were operated by a LC system, LC-20 Prominence (Shimadzu Co.). LC-MS analyses for IgG were operated by a LC system, LC-20 Prominence XR (Shimadzu Co.), a mass spectrometer, Q-TOF premier (Waters Co.), and a protein analysis soft, MassLynxs V4.1 (Waters Co.). LC-MS analyses for the digestion with Pepsin were operated by a LC system, LC1100 or LC 1200 (Agilent Technologies), a mass spectrometer, LTQ/XL Orbitrap (Thermo Fisher Scientific), and a peptide mapping soft, PepFinder 2.0 (Thermo Fisher Scientific).

MS conditions with a Q-TOF premier were follows: capillary, 3.5 kV; sampling cone, 80.0 V; source temperature, 100 °C; cone gas flow, 50.0 (L/Hr), and with a LTQ/XL Orbitrap were follows: spray voltage, 4 kV; capillary temperature, 325 °C; capillary voltage, 10 V.

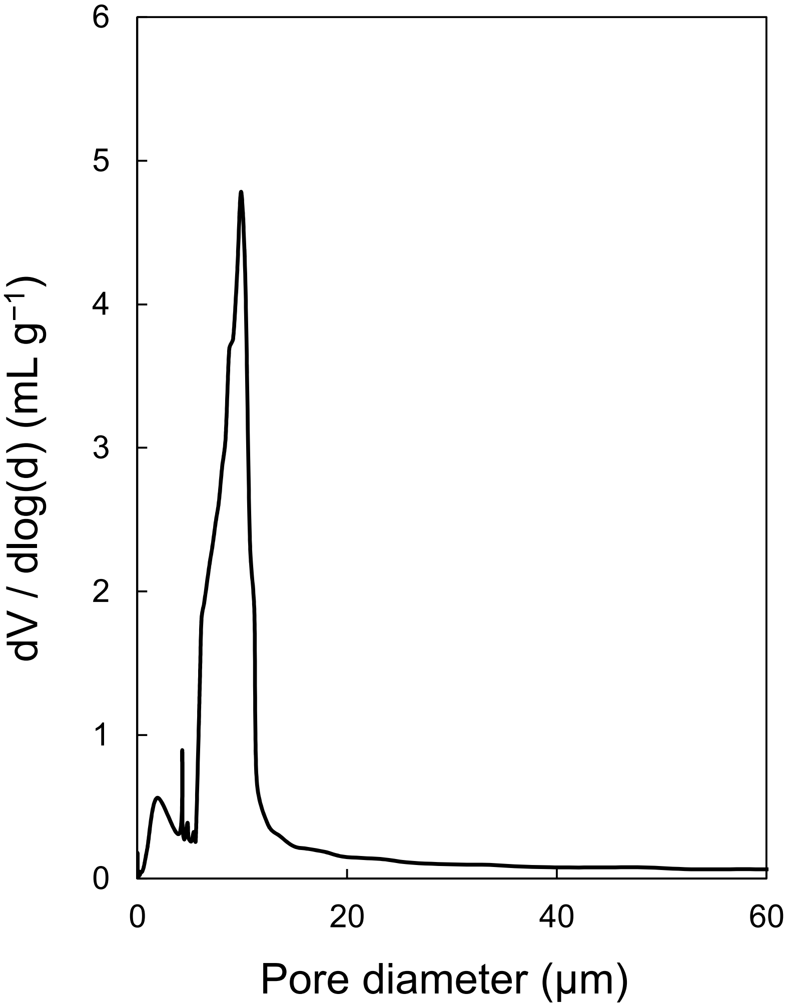

Supplementary Fig. 1. Pore distribution of a PEGM-based spongy monolith by mercury porosimetry.

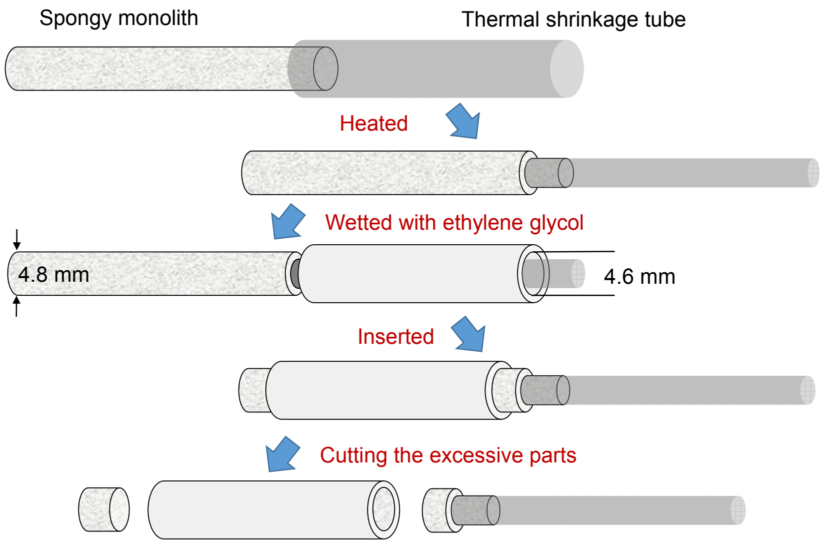

Supplementary Fig. 2. Schematics of the packing procedures of spongy monoliths.

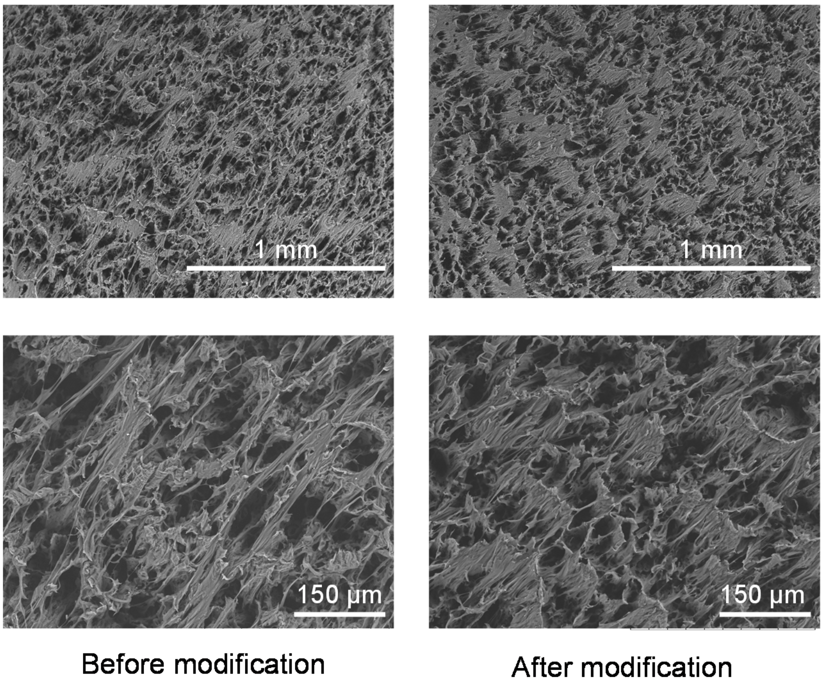

Supplementary Fig. 3. SEM images of the spongy monolith before and after immobilization with Protein A.

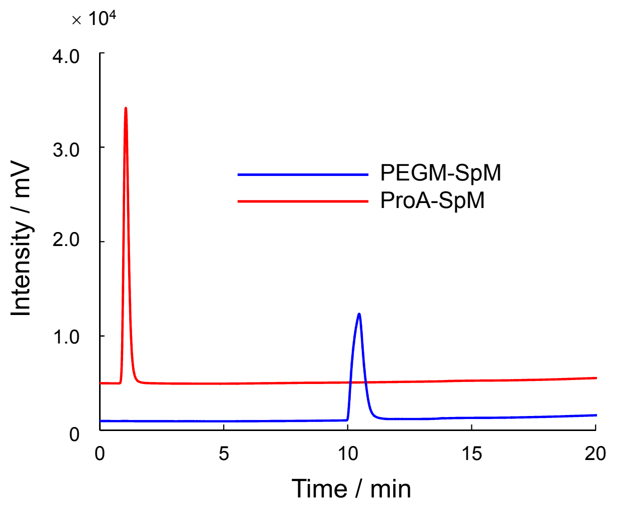

Supplementary Fig. 4. Chromatograms of BSA with PEGM-SpM or ProA-SpM.

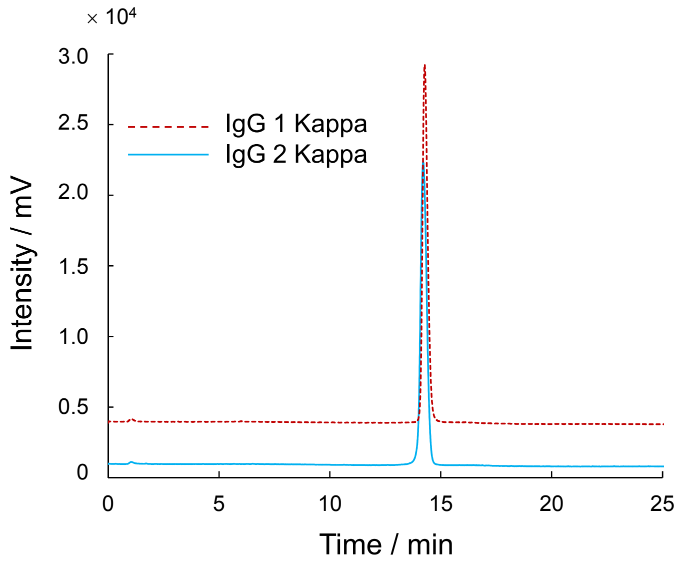

Supplementary Fig. 5. Chromatograms of IgG standards with ProA-SpM by pH gradient.

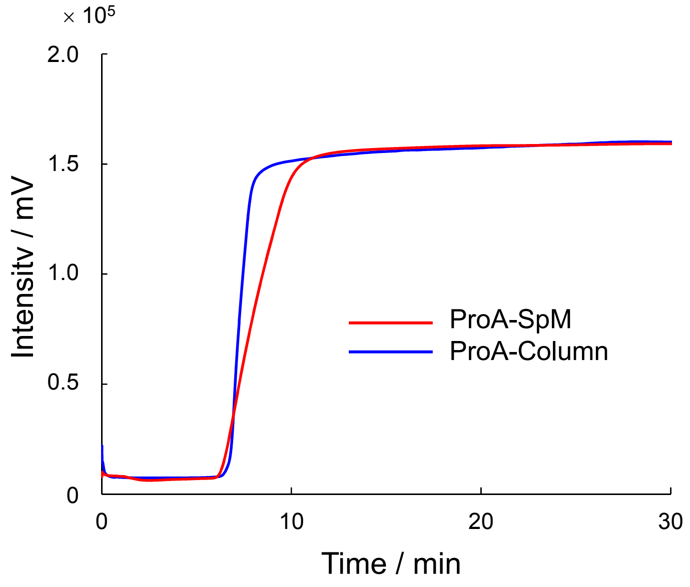

Supplementary Fig. 6. Frontal analyses using IgG standard with ProA-Column and ProA-SpM.

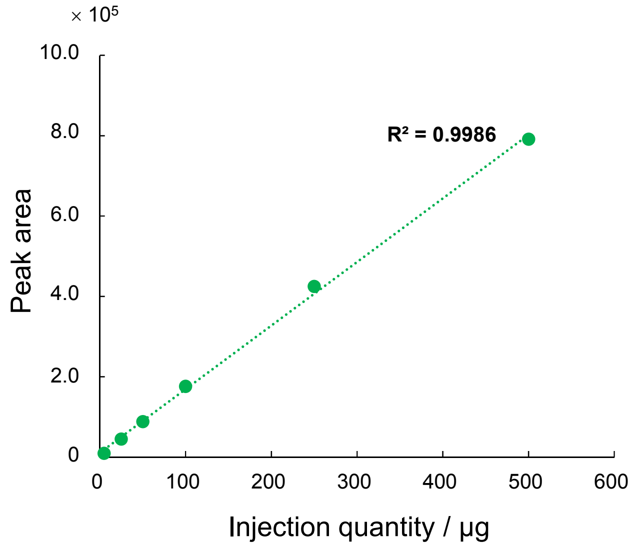

Supplementary Fig. 7. Linearity of injected IgG under rapid elution, 9.0 mL min^{-1}.

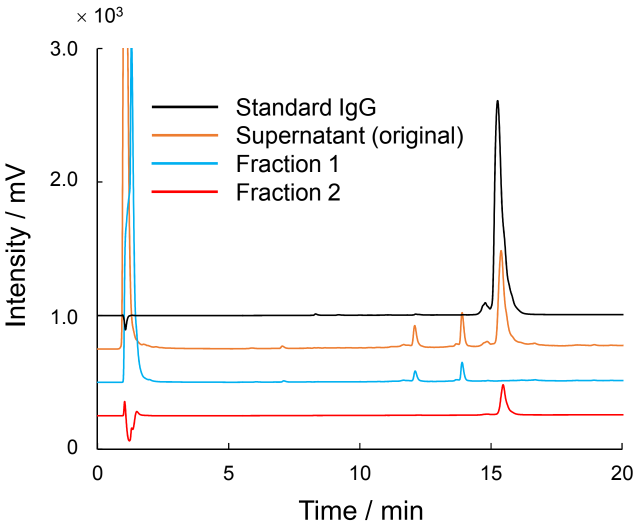

Supplementary Fig. 8. The UV chromatograms of a Protein A load sample.

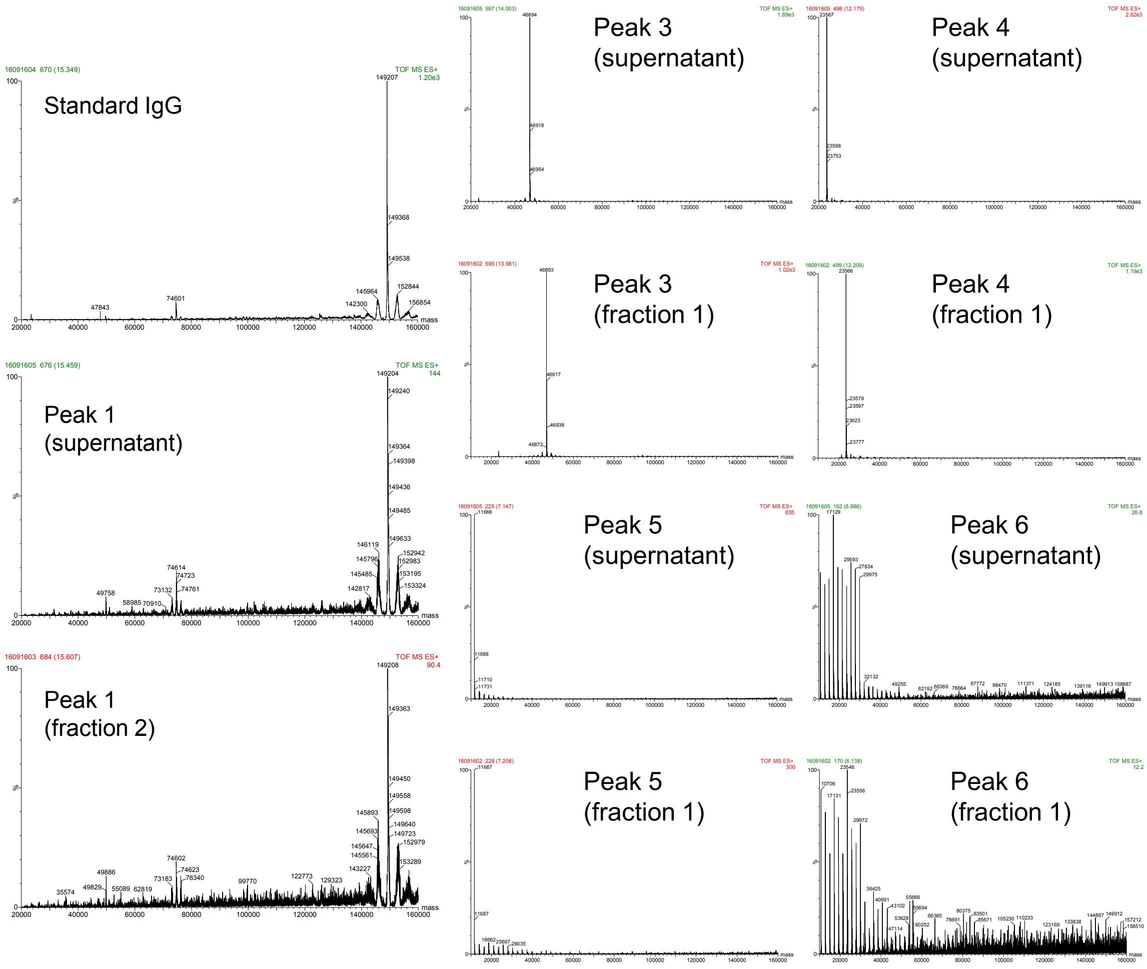

Supplementary Fig. 9. MS spectra of each peak by detected from a recombinant human IgG1 antibody. The peaks are corresponding to Fig. 1(k).

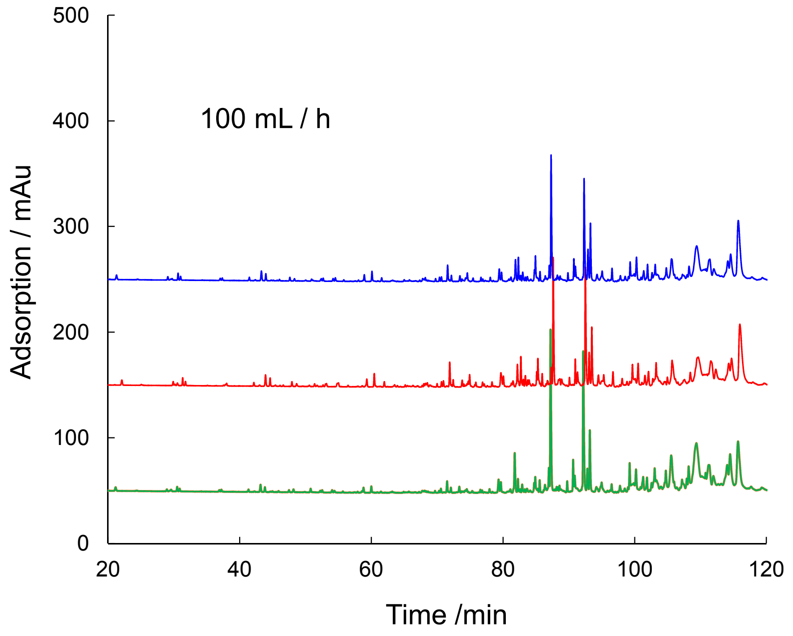

Supplementary Fig. 10. The UV chromatograms for repeatability of online digestion with Pep-SpM under 100 mL h^{-1} as flow rate.

Supplementary Table 1. Assigned peptides by online digestion of a recombinant human IgG1 antibody with Pep-SpM. These peptides were assigned by PepFinder 2.0. based on all the peptides detected by LC-MS containing the origin (light or heavy chain). The number of amino acids of each terminal and the length are summarized. Here, the number of amino acid was assigned that the number 1 is first amino acid from N-terminal.

Flow Rate 100 mL h^{-1}							Flow Rate 10 mL h^{-1}						
Retention Time (min)	Identified peptide						Retention Time (min)	Identified peptide					
	Chain	N-terminal		C-terminal		Length		Chain	N-terminal		C-terminal		Length
16.2563	L	Y	87	Q	90	4	16.2486	L	Y	87	Q	90	4
25.186	L	K	125	V	131	7	25.1773	L	K	125	V	131	7
25.4079	H	Q	177	L	181	5	25.3485	H	Q	177	L	181	5
30.0255	L	Y	91	Y	94	4	26.2261	L	S	161	T	171	11
30.2895	L	F	83	T	85	3	30.024	L	Y	91	Y	94	4
30.6824	H	D	401	F	406	6	30.2692	L	F	83	T	85	3
31.4681	L	Y	87	S	93	7	30.625	H	D	401	F	406	6
31.884	L	Y	86	Q	90	5	31.4988	L	Y	87	S	93	7
35.2608	H	I	379	E	382	4	31.8938	L	Y	86	Q	90	5
37.6997	L	T	5	L	11	7	35.2807	H	I	379	E	382	4
37.7475	L	K	148	E	160	13	37.7173	L	T	5	L	11	7
38.1499	L	A	84	Q	90	7	37.9938	L	A	84	Y	87	4
42.2943	L	Y	86	S	93	8	38.3718	H	Y	182	S	186	5
43.1654	L	D	1	M	4	4	39.3537	L	Q	123	V	131	9
44.0371	L	F	83	Y	86	4	41.7937	H	I	255	T	262	8

44.7465	L	Y	87	Y	94	8	42.3163	L	Y	86	S	93	8
45.4826	L	V	132	L	134	3	43.8953	L	I	75	D	82	8
45.5874	H	V	264	D	272	9	44.0758	L	F	83	Y	86	4
48.0523	L	Y	36	L	46	11	44.4621	H	V	5	S	17	13
48.7965	H	I	255	C	263	9	44.487	H	T	69	L	79	11
50.6965	L	L	47	Y	49	3	44.7377	H	Y	94	M	103	10
51.5222	H	V	371	D	378	8	44.7598	L	Y	87	Y	94	8
52.798	L	T	179	E	194	16	45.4787	L	V	132	L	134	3
53.3235	L	F	83	Q	90	8	45.6127	H	V	264	D	272	9
54.9483	L	F	83	Y	87	5	46.1097	H	E	1	L	4	4
55.1311	L	L	135	E	142	8	46.2323	H	N	84	V	93	10
58.0703	H	I	255	V	264	10	46.2734	L	S	181	E	194	14
59.2466	H	L	370	D	378	9	48.0088	L	Y	36	L	46	11
59.4285	L	D	1	L	11	11	48.2478	L	T	74	D	82	9
60.5378	L	V	195	C	213	19	48.6997	H	I	255	C	263	9
62.1063	L	A	143	E	160	18	48.8763	L	S	161	T	177	17
62.1547	L	K	24	W	35	12	50.722	L	L	47	Y	49	3
62.5981	L	D	1	D	17	17	51.4312	H	V	371	D	378	8
63.453	L	K	125	L	134	10	51.8283	L	S	12	T	22	11
63.6346	H	V	429	G	448	20	51.8554	L	A	143	W	147	5
64.09	H	T	413	F	425	13	52.3511	H	E	359	L	367	9
64.5711	L	S	161	L	178	18	52.6215	H	E	1	L	4	4

65.308	L	R	95	V	114	20	52.769	L	T	179	E	194	16
67.1361	L	T	72	D	82	11	52.9656	H	V	188	T	199	12
67.854	L	V	33	L	46	14	54.1503	L	F	71	T	74	4
68.1168	L	Y	94	V	114	21	55.0588	L	W	50	D	70	21
68.4145	H	W	383	L	400	18	55.0588	H	M	254	C	263	10
68.6245	L	S	181	C	213	33	55.2225	H	Y	182	V	187	6
69.0118	H	F	407	L	412	6	55.496	L	V	131	L	134	4
70.125	L	Y	91	V	114	24	56.5512	H	V	187	T	199	13
70.7921	L	T	179	C	213	35	58.0617	H	I	379	N	392	14
71.1183	H	S	426	G	448	23	58.0617	H	I	255	V	264	10
71.6	H	V	371	E	382	12	58.8873	L	S	12	C	23	12
72.026	L	L	47	D	70	24	59.2177	H	L	370	D	378	9
72.2769	L	K	24	L	46	23	59.3608	H	H	431	G	448	18
72.6228	L	Y	87	V	114	28	59.3608	L	D	1	L	11	11
73.8955	H	W	383	F	406	24	60.5088	L	V	195	C	213	19
73.9915	L	C	23	L	46	24	60.7397	L	L	178	E	194	17
74.2131	L	L	178	C	213	36	61.4625	L	E	194	C	213	20
74.6945	L	R	95	F	115	21	61.6085	H	V	429	K	449	21
74.8552	L	D	1	T	22	22	62.0635	L	A	143	E	160	18
74.8943	L	Y	86	V	114	29	63.689	H	V	429	G	448	20
75.0746	L	L	135	E	160	26	63.689	L	C	23	W	35	13
75.848	L	A	84	V	114	31	64.0969	H	T	413	F	425	13

75.989	L	S	161	E	194	34	64.2954	H	L	237	F	243	7
77.028	L	Y	94	F	115	22	64.5605	L	S	161	L	178	18
77.2946	H	Y	182	T	199	18	64.9275	H	T	413	S	428	16
78.4786	L	Y	91	F	115	25	65.261	L	R	95	V	114	20
79.0127	L	D	1	C	23	23	65.5468	H	E	1	S	17	17
79.8161	H	I	379	L	400	22	65.5468	H	T	309	E	320	12
79.9533	L	L	47	F	71	25	66.9422	H	V	371	A	380	10
80.2163	L	Y	87	F	115	29	67.0433	L	T	72	D	82	11
81.6356	L	F	71	D	82	12	67.0433	H	V	148	T	157	10
81.7403	L	V	132	E	142	11	67.1793	H	S	428	G	448	21
82.128	L	Y	86	F	115	30	67.8288	L	V	33	L	46	14
82.358	H	I	379	F	406	28	68.0648	L	Y	94	V	114	21
82.8493	L	S	161	C	213	53	68.3037	H	W	383	L	400	18
83.4791	L	S	170	C	213	44	68.591	L	S	181	C	213	33
83.5848	L	T	72	F	83	12	68.591	L	A	32	L	46	15
83.5848	L	S	12	L	46	35	68.9706	H	F	407	L	412	6
83.813	H	F	407	F	425	19	69.0472	H	S	426	K	449	24
84.1388	L	Y	36	D	70	35	69.1245	H	T	368	D	378	11
84.656	L	L	47	T	74	28	69.2903	H	Y	200	L	236	37
85.5998	L	W	50	D	82	33	69.697	H	Y	393	F	406	14
85.8136	H	Y	409	K	449	41	70.0908	L	Y	91	V	114	24
86.1053	H	T	413	G	448	36	70.7877	L	T	179	C	213	35

86.6843	L	F	83	F	115	33	71.0471	H	S	426	G	448	23
87.4643	H	Y	409	G	448	40	71.4985	H	Y	409	F	425	17
88.6947	L	V	132	E	160	29	71.6321	H	V	371	E	382	12
89.3583	L	R	95	V	131	37	71.8381	H	L	147	T	157	11
90.4489	L	Y	94	V	131	38	71.9752	L	L	47	D	70	24
90.8482	L	Y	91	V	131	41	72.4119	H	E	359	L	370	12
91.0745	H	F	407	K	449	43	72.6163	L	Y	87	V	114	28
91.4385	L	D	1	L	46	46	72.6163	H	I	104	L	114	11
91.6291	L	Y	87	V	131	45	72.7808	H	V	381	L	400	20
92.645	L	F	83	L	124	42	73.914	H	W	383	F	406	24
92.645	H	V	371	F	406	36	73.9905	L	C	23	L	46	24
93.2428	L	L	47	D	82	36	74.2305	L	L	178	C	213	36
94.6313	L	W	50	F	83	34	74.6902	L	R	95	F	115	21
94.6313	L	F	71	F	83	13	74.8489	L	L	135	W	147	13
94.6313	H	V	158	L	176	19	74.8985	L	Y	86	V	114	29
95.4596	L	S	12	D	70	59	74.8985	L	D	1	T	22	22
96.3011	L	W	50	Y	86	37	75.8386	L	Y	172	E	194	23
96.7856	L	F	71	Y	86	16	75.8798	L	A	84	V	114	31
96.8625	L	Y	36	D	82	47	76.0312	L	S	161	E	194	34
98.8544	L	R	95	L	134	40	76.3668	H	L	370	E	382	13
99.4037	L	L	47	T	85	39	76.4683	H	E	1	L	18	18
99.5636	L	Y	94	L	134	41	77.0042	L	Y	94	F	115	22

99.7238	L	Y	91	L	134	44	77.0748	H	D	401	L	408	8
100.019	L	Y	87	L	134	48	77.1763	H	T	166	L	176	11
100.229	H	Y	200	M	254	55	77.279	H	Y	182	T	199	18
100.66	L	L	47	F	83	37	77.279	H	P	191	T	199	9
101.745	L	L	47	Y	86	40	77.6782	L	S	161	L	180	20
102.219	L	Y	36	F	83	48	77.7542	L	L	135	T	177	43
102.878	L	Y	36	Y	86	51	78.4331	L	Y	91	F	115	25
104.821	H	V	148	L	176	29	78.498	H	Y	200	V	242	43
							78.6612	L	L	47	F	62	16
							79.0015	L	D	1	C	23	23
							79.1568	H	T	309	E	335	27
							79.2615	H	V	115	L	144	30
							79.5169	H	V	115	C	146	32
							79.8138	H	I	379	L	400	22
							79.9655	L	L	47	F	71	25
							80.2291	L	Y	87	F	115	29
							80.8587	H	T	309	V	350	42
							81.2565	H	Y	182	Y	200	19
							81.4668	H	V	264	W	279	16
							81.549	H	Y	182	L	195	14
							81.7878	L	V	132	E	142	11
							82.1408	L	Y	86	F	115	30

							82.3517	H	I	379	F	406	28
							82.5123	L	S	167	C	213	47
							82.8535	L	S	161	C	213	53
							83.243	H	F	407	S	428	22
							83.5777	L	S	12	L	46	35
							83.5777	L	T	72	F	83	12
							83.8712	H	V	264	G	283	20
							83.8712	H	F	407	F	425	19
							84.0317	L	Y	36	D	70	35
							84.3237	H	T	413	K	449	37
							84.6498	L	L	47	T	74	28
							85.0453	H	T	309	C	369	61
							85.0453	H	T	309	L	367	59
							85.2045	L	L	135	E	194	60
							85.5982	L	W	50	D	82	33
							85.7765	H	Y	409	K	449	41
							86.0538	H	T	413	G	448	36
							87.0462	H	Y	200	F	243	44
							87.2253	H	Y	182	L	236	55
							87.4913	H	Y	409	G	448	40
							87.7194	L	L	47	L	73	27
							88.5248	L	T	5	L	46	42

							88.5248	L	Y	36	F	71	36
							88.687	L	V	132	E	160	29
							88.9361	H	V	115	L	147	33
							89.2455	L	R	95	V	131	37
							90.3494	L	Y	94	V	131	38
							90.8672	L	Y	91	V	131	41
							91.059	H	F	407	K	449	43
							91.374	L	D	1	L	46	46
							91.5013	L	Y	87	V	131	45
							92.2118	H	V	371	L	400	30
							92.2118	L	V	132	W	147	16
							92.637	H	V	371	F	406	36
							93.1772	L	L	47	D	82	36
							94.5832	L	W	50	F	83	34
							94.5832	H	V	158	L	176	19
							94.5832	L	F	71	F	83	13
							94.7828	H	L	370	F	406	37
							95.3685	L	S	12	D	70	59
							96.8033	L	Y	36	D	82	47
							97.4023	H	V	158	L	181	24
							98.197	H	Y	200	L	253	54
							98.7978	L	R	95	L	134	40

							99.2843	H	N	203	M	254	52
							99.479	L	Y	94	L	134	41
							100.058	H	Y	200	M	254	55
							100.639	L	L	47	F	83	37
							102.965	H	V	115	T	157	43
							104.781	H	V	148	L	176	29
							105.764	H	V	148	L	181	34
							106.385	H	L	147	L	176	30
							107.125	H	L	147	L	181	35
							108.022	H	V	158	T	199	42
							108.586	H	G	145	L	176	32
							109.074	H	G	145	L	181	37
							112.548	H	V	148	T	199	52
